# Supplementary material for: Validity of the Actigraph-GT9X accelerometer for measuring steps and energy expenditures in heart failure patients
Source: PLoS One. 2024 Dec 30;19(12):e0315575. doi: 10.1371/journal.pone.0315575 (PMC11684600; doi:10.1371/journal.pone.0315575)
Supplement: S1 Table — (DOCX) [file pone.0315575.s001.docx]

**Supporting information**

| **S1 Table. Glossary of abbreviations.** | |
| --- | --- |
| Abbreviations | Definition |
| AG | ActiGraph |
| HFpEF | Heart Failure with preserved Ejection Fraction |
| SC | Step Counts |
| EE | Energy Expenditure |
| VT | Vertical axis |
| VM | Vector Magnitude |
| METs | Metabolic Equivalents |
| MAPE | Mean Absolute Percentage Error |

Abbreviations and definitions used in the current study.
