# Supplementary material for: Validity of the Actigraph-GT9X accelerometer for measuring steps and energy expenditures in heart failure patients
Source: PLoS One. 2024 Dec 30;19(12):e0315575. doi: 10.1371/journal.pone.0315575 (PMC11684600; doi:10.1371/journal.pone.0315575)
Supplement: S2 Table — (DOCX) [file pone.0315575.s002.docx]

**Supporting information**

| **S2 Table. Bland-Altman analysis for step counts and energy expenditure in each location and algorithm.** | | | | | | | |
| --- | --- | --- | --- | --- | --- | --- | --- |
|  | Location/Algorithms | *b* | S.E | *p* | Mean bias | 95% LOA | |
|  |  |  |  |  |  | Lower | Upper |
| Step Counts | Ankle SC | -0.06 | 0.02 | .006 | -0.98 | -5.80 | 3.83 |
|  | Waist SC | -0.50 | 0.03 | <.0001 | -4.27 | -12.54 | 4.00 |
| Energy Expenditure | Freedson | -0.21 | 0.03 | <.0001 | -0.36 | -1.40 | 0.69 |
|  | Freedson Combination | 0.01 | 0.05 | 0.878 | -0.42 | -2.17 | 1.33 |
|  | Refined Crouter (10sec) | -0.56 | 0.01 | <.0001 | -0.18 | -1.39 | 1.03 |
|  | Refined Crouter (60sec) | -0.54 | 0.02 | <.0001 | -0.19 | -1.23 | 0.86 |
|  | Sasaki | 0.16 | 0.04 | .0002 | 0.02 | -1.50 | 1.54 |
|  | Santos-Lozano VT | -0.33 | 0.03 | <.0001 | 0.16 | -0.74 | 1.06 |
|  | Santos-Lozano VM | -0.37 | 0.02 | <.0001 | 0.14 | -0.87 | 1.14 |

SC = Step Counts; S.E = Standard Error; LOA = Limit of Agreement; VT = vertical axis; VM = Vector Magnitude.
