# Supplementary material for: Validity of the Actigraph-GT9X accelerometer for measuring steps and energy expenditures in heart failure patients
Source: PLoS One. 2024 Dec 30;19(12):e0315575. doi: 10.1371/journal.pone.0315575 (PMC11684600; doi:10.1371/journal.pone.0315575)
Supplement: S3 Table — (DOCX) [file pone.0315575.s003.docx]

**Supporting information**

| **S3 Table. Total Step Counts and Energy Expenditure During Cardiopulmonary Treadmill Test Using Raw Data.** | | | |
| --- | --- | --- | --- |
|  | Overall^a^ | LPA^a^ | MVPA^a^ |
| **Criterion measure** |  |  |  |
| CPET duration (minutes) | 9.93 (8.74–11.13) | 4.97 (4.38–5.55) | 5.23 (4.63–5.82) |
| VO_2_ ml/kg/min | 10.53 (9.79–11.27) | 7.77 (7.38–8.15) | 13.23 (12.84–13.62) |
| METs | 3.01 (2.80–3.22) | 2.22 (2.11–2.33) | 3.78 (3.67–3.89) |
| Manual step counts | 901.00 (769.44–1032.57) | 400.28 (337.45–463.12) | 527.87 (464.10–591.63) |
| **ActiGraph GT9X** |  |  |  |
| **Total Steps (counts)** |  |  |  |
| Ankle-worn | 851.31 (736.73–965.88)* | 368.19 (309.29–427.10)* | 516.06 (455.82–576.29)* |
| Waist-worn | 653.37 (517.26–789.48) | 213.46 (148.84–278.07) | 457.66 (392.16–523.16) |
| **Energy expenditure (METs)^c^** |  |  |  |
| Freedson | 2.74 (2.48–3.00) | 2.19 (1.99–2.39)* | 3.30 (3.10–3.50) |
| Freedson Combination | 2.63 (2.23–3.03) | 1.89 (1.56–2.22) | 3.38 (3.05–3.72) |
| Refined Crouter (10sec) ^b^ | 2.86 (2.68–3.03)* | 2.53 (2.40–2.67) | 3.16 (3.03–3.30) |
| Refined Crouter (60sec) ^b^ | 2.90 (2.74–3.07)* | 2.59 (2.46–2.72)* | 3.23 (3.10–3.36) |
| Sasaki | 3.14 (2.76–3.51)* | 2.35 (2.05–2.66)* | 3.93 (3.63–4.24)* |
| Santos-Lozano VT | 3.26 (3.09–3.43)* | 2.80 (2.68–2.92) | 3.74 (3.61–3.86)* |
| Santos-Lozano VM | 3.24 (3.07–3.42)* | 2.82 (2.68–2.96) | 3.68 (3.53–3.82)* |
| LPA = Light-intensity Physical Activity (1.50−2.99 METs); MVPA = Moderate-to-Vigorous Physical Activity (≥3 METs); VT = Vertical Axis; VM = Vector Magnitude.  ^a^Values are presented as mean (95% confidence intervals) from a random effect mixed model.  ^b^The Refined Crouter equation used both 10 sec- and 60 sec-epoch data to estimate energy expenditure.  ^c^AG-derived EEs were predicted from waist-worn AG accelerometers.  ^*^ Significantly equivalent with the criterion measure at 10% equivalence zone based on two one-sided *t*-tests equivalence test (*p*<0.05). Raw data did not exclude 5% of data at the beginning and the end of the CPET. | | | |
