# Supplementary material for: Validity of the Actigraph-GT9X accelerometer for measuring steps and energy expenditures in heart failure patients
Source: PLoS One. 2024 Dec 30;19(12):e0315575. doi: 10.1371/journal.pone.0315575 (PMC11684600; doi:10.1371/journal.pone.0315575)
Supplement: S4 Table — (DOCX) [file pone.0315575.s004.docx]

**Supporting information**

| **S4 Table. Accuracy and Agreement of AG-Derived Step Counts and Energy Expenditures Compared to Criterion Measures Using Raw Data.** | | | | | |
| --- | --- | --- | --- | --- | --- |
|  | MAPE (%)^a^ | % bias ^a^ | Mean bias | 95% LOA | |
|  |  |  |  | Lower | Upper |
| **Step counts** |  |  |  |  |  |
| **Ankle SC** |  |  |  |  |  |
| Overall | 10.50 (7.14–13.87) | -7.11 (-10.95– -3.28) | -1.15 | -6.17 | 4.73 |
| LPA | 12.53 (9.32–15.74) | -10.40 (-14.15– -6.65) | -1.56 | -7.12 | 4.00 |
| MVPA | 8.36 (5.14–11.57) | -3.63 (-7.38–0.13) | -0.76 | -6.00 | 4.48 |
| **Waist SC** |  |  |  |  |  |
| Overall | 32.66 (25.03–40.28) | -31.26 (-39.23– -23.29) | -4.41 | -12.84 | 4.03 |
| LPA | 47.84 (41.85–53.83) | -47.60 (-53.93– -41.26) | -6.37 | -14.60 | 1.85 |
| MVPA | 17.10 (11.11–23.10) | -14.54 (-20.87– -8.20) | -2.57 | -9.66 | 4.52 |
| **Energy expenditure** |  |  |  |  |  |
| **Freedson** |  |  |  |  |  |
| Overall | 17.04 (14.88–19.21) | -8.56 (-14.00– -3.11) | -0.33 | -1.45 | 0.79 |
| LPA | 17.46 (15.09–19.84) | -3.77 (-9.17–1.64) | -0.17 | -1.07 | 0.74 |
| MVPA | 16.61 (14.23–18.99) | -18.97 (-18.97– -8.12) | -0.52 | -1.67 | 0.63 |
| **Freedson Combination** |  |  |  |  |  |
| Overall | 30.21 (23.81–36.60) | -16.27 (-29.10– -3.44) | -0.45 | -2.22 | 1.32 |
| LPA | 44.71 (38.61–50.81) | -19.76 (-31.99– -7.53) | -0.53 | -2.67 | 1.61 |
| MVPA | 15.17 (9.05–21.29) | -12.65 (-24.91– -0.38) | -0.34 | -1.70 | 1.02 |
| **Refined Crouter (10sec)** ^b^ | |  |  |  |  |
| Overall | 23.05 (20.95–25.16) | 4.03 (-0.83–8.89) | -0.16 | -1.65 | 1.32 |
| LPA | 29.12 (26.69–31.55) | 23.44 (19.14–27.74) | 0.33 | -0.67 | 1.33 |
| MVPA | 16.86 (14.41–19.30) | -16.02 (-20.34– -11.70) | -0.55 | -1.58 | 0.48 |
| **Refined Crouter (60sec)** ^b^ | |  |  |  |  |
| Overall | 17.10 (15.01–19.18) | 0.20 (-3.96–4.35) | -0.16 | -1.40 | 1.07 |
| LPA | 18.82 (16.15–21.48) | 14.54 (10.28–18.80) | 0.25 | -0.54 | 1.05 |
| MVPA | 15.37 (12.72–18.02) | -14.46 (-18.74– -10.18) | -0.56 | -1.55 | 0.43 |
| **Sasaki** |  |  |  |  |  |
| Overall | 19.05 (13.98–24.11) | 2.47 (-7.59–12.54) | 0.05 | -1.48 | 1.59 |
| LPA | 21.87 (16.74–26.99) | 2.99 (-6.57–12.56) | 0.01 | -1.38 | 1.40 |
| MVPA | 16.09 (10.95–21.24) | 1.93 (-7.66–11.51) | 0.06 | -1.53 | 1.65 |
| **Santos-Lozano VT** |  |  |  |  |  |
| Overall | 17.17 (14.08–20.26) | 11.30 (7.38–15.22) | 0.19 | -0.86 | 1.24 |
| LPA | 24.76 (21.42–28.09) | 23.63 (19.83–27.43) | 0.44 | -0.36 | 1.24 |
| MVPA | 9.53 (6.19–12.87) | -1.11 (-4.92–2.70) | -0.07 | -1.01 | 0.87 |
| **Santos-Lozano VM** |  |  |  |  |  |
| Overall | 18.86 (15.22–22.50) | 11.56 (5.08–18.03) | 0.17 | -0.97 | 1.32 |
| LPA | 27.48 (23.78–31.18) | 25.14 (19.57–30.71) | 0.49 | -0.45 | 1.42 |
| MVPA | 10.18 (6.46–13.89) | -2.41 (-8.00–3.18) | -0.13 | -1.06 | 0.79 |
| AG = ActiGraph; SC = Step Counts; MAPE = Mean Absolute Percentage Error; LOA = Limit of Agreement; LPA = Light-intensity Physical Activity (1.50−2.99 METs); MVPA = Moderate-to-Vigorous Physical Activity (≥3 METs); VT = Vertical axis; VM = Vector Magnitude.  ^a^ Values are presented as mean (95% confidence intervals).  ^b^ The Refined Crouter equation used both 10 sec- and 60 sec-epoch data.  Raw data did not exclude 5% of data at the beginning and the end of the CPET. | | | | | |
