# Supplementary material for: Validity of the Actigraph-GT9X accelerometer for measuring steps and energy expenditures in heart failure patients
Source: PLoS One. 2024 Dec 30;19(12):e0315575. doi: 10.1371/journal.pone.0315575 (PMC11684600; doi:10.1371/journal.pone.0315575)
Supplement: Attachment — Submitted filename: R1_Response to Reviewers.docx [file pone.0315575.s006.docx]

Review Comments to the Author

**Reviewer #1**: A research study was conducted that examined the criterion validity of the AG-GT9X for measuring step counts (SC) and energy expenditure (EE) among heart failure patients. Data from some patients was repeatedly measured: 41 CPET from 16 participants. The results are unclear because the statistical analysis is inappropriate for analyzing repeated-measures data.

Major revisions:
Overall, a comprehensive re-analysis of the repeated measures data is needed.
1- Since data collection occurred repeatedly for some participants the analysis should be tailored for repeated measures data. Thus Pearson’s correlation and t-tests are not appropriate in a repeated-measure study as it ignores the correlation of the outcomes from multiple visits within the same participant. Furthermore, the calculation of standard deviation deviates from the conventional method when repeated measures are performed.

*1-Response: Thank you for your valuable comments. To address your comments, we re-analyzed the overall study outcomes using appropriate statistical methods, considering the structure of the repeated measures data. We re-calculated correlation coefficients between criterion-derived and AG-derived measures using a mixed model with a random intercept to account for multiple observations within each subject. Additionally, we used the bootstrapping methods by replicating 200 bootstrap samples at the individual level to estimate the bias-corrected 95% confidence intervals.*

2- The standard statistical term for average is mean.

*2-Response: We revised the statistical terms ‘average’ to ‘mean’ throughout the manuscript.*

3- Table 1: For continuous data, if it is normally distributed, summarize as means and standard deviations. If the distribution of the data violates the normality assumption, summarize as medians, first and third quartiles.

*3-Response: We revised Table 1 in accordance with your comment. We also included additional clinical information and biomarkers, ensuring that they are described with appropriate units after checking distribution assumptions were met.*

**Reviewer #2:** Thank you for submitting this work.

The manuscript is validating the use of the ActiGraph accelerometer to assess physical activity in heart failure patients. They have examined step counts and energy expenditure using this accelerometer. They found out that this accelerometer successfully showed better accuracy in laboratory settings but need to be validated in other clinical settings.

Body of manuscript is written good. No further comments to revise it.

**Reviewer #3**: General Comments:

PONE-D-24-20379 presents a validation of device-based energy expenditure estimation methods and step counts in a sample of patients with heart failure. There are several areas that need attention. Please see specific comments and suggestions by section of the manuscript below.

The references have some inconsistencies. Please complete a thorough check.

*Response: We completed a thorough check and addressed inconsistencies in our references.*

**Abstract**:

Is the Total # here the number of patients or the number of tests? Please consider clarifying. I encourage the authors to present the N of patients (mean age +/- sd) and then perhaps the median (IQR) number of tests. I find that more relevant than the total number of tests.

Which ankle was the GT9X worn on? Please consider specifying if adequate space is available

*Response: Thanks for your valuable suggestions. We revised the abstract by including additional information based on your comments. – lines 36-40: “16 patients with HF with preserved ejection fraction (mean age=60.3±12.1yrs) completed a total of 41 symptom-limited cardiopulmonary exercise tests on a treadmill across multiple time points (median (IQR)=2.5 (1.5–3.5)). All participants wore the AG (model: GT9X) on both the right ankle and waist locations during the test.”*

**Introduction**:
• Lines 17-18: please consider revising this to reflect that ActiGraph is a device manufacturer and that there are multiple device models available. “The Actigraph” reads to me as if it is a singular device type which may be misleading for some readers.

*Response: Valid point. We revised ‘The Actigraph’ to ‘The Actigraph-GT9X’ to clearly identify it as a specific device model of the accelerometer, ensuring clarity for readers and avoiding any potential confusion – lines 71-72: “The Actigraph (AG)-GT9X (ActiGraph Inc, Pensacola, FL, USA) is one of the commonly used accelerometer devices to assess physical activity measures.”*

• Lines 18-22: please consider revising for clarity. The devices collect high temporal resolution triaxial acceleration that is commonly summarized by calculating a vector magnitude. This is not limited to just habitual locomotor activities. Similar to my comment above, I encourage the authors to be explicit for readers that may not be as familiar with the device-based literature. It may also be worthwhile to mention how counts factor in to this as that may not be understood by all.

*Response: Thank you for your insightful comments. We revised the overall paragraph to enhance clarity and added context about how acceleration data is processed into activity counts to derive physical activity indicators. – lines 72-77: “The AG-GT9X is a triaxial accelerometer that can measure acceleration in three individual orthogonal planes (i.e., vertical (VT), anteroposterior, and mediolateral) [10, 11]. It summarizes high temporal resolution triaxial acceleration data by calculating the vector magnitude (VM) to provide a comprehensive measure of human behavior and movement [12]. Acceleration data can be processed into activity counts, which can be used to obtain indicators to assess PA levels* *[13].”*

• Lines 22-26: similar to my above comments, I encourage the authors to give more context about these summary measures derived from the acceleration data. Steps and EE are just two of many device-based PA outcomes to be obtained from these data. I think this paragraph could benefit from additional detail.

*Response: We included more context about summarized high-resolution triaxial acceleration data from the device and acceleration data processing into activity counts to derive physical activity indicators, particularly steps and energy expenditure, which are mainly focused on in the current study. – lines 77-82: “Step counts (SC) and estimated energy expenditure (EE) are commonly used indicators to quantify PA levels utilizing activity counts derived from acceleration data [10, 14-16]. SC is a simple and objective measure of activity counts with higher values indicating a greater amount of activity; while EE provides a more complex measure of PA intensity levels that reflects the amount of energy expended during PA.”*

• Lines 27-30: this sentence may be enhanced by adding something about the outcome of interest also being a factor. This may make it more clear to the reader why the ankle is generally better for steps and the waist for EE, etc.

*Response: Thank you for your valuable suggestion. We anticipated that the first sentence in this paragraph would comprehensively reflect the current study's outcome of interest, followed by the sentences highlighting the variations in steps across different wear locations, and the following paragraph highlighting variations in EE estimation across different equations, which are only developed using waist-worn AG among different population groups (e.g., young healthy adults, older adults) or different environmental settings (e.g., laboratory, free-living). To improve clarity, we revised the sentences to more accurately reflect the study’s outcome of interest –* *lines 83-87: “Nevertheless, accurately assessing PA levels with AG is challenging due to several factors, including variations in SC across the placement of AG on the body (i.e., wrist, ankle, or waist), environment settings (i.e., laboratory or free-living), population demographics (i.e., younger or older adults), and the use of different prediction equations to estimate EE developed relying on waist-worn AG and targeting different population groups.”*

• Lines 31-34: Ref 16 duplicated. Please check.

*Response: Thank you for pointing out the error. We deleted the duplicated reference from this sentence.*

• Lines 41-47: I would consider adding in here that these and many of the EE equations were specifically developed using data from waist-worn devices.

*Response: Valid point. We added this context in paragraph – lines 102-105: “**However, these equations were specifically developed using data obtained from waist-worn AG and mainly targeting young and middle-aged adults, which made their validity in predicting EE subject to debate depending on the PA types, intensity, or population groups”*

• Lines 64-66: should this be “indirect calorimetry measured EE”?

*Response: Good point. We revised ‘indirectly measured EE’ to ‘indirect calorimetry-measured EE’. -lines 122-124: “Specifically, this study compared the AG-derived SC with manually counted steps and evaluated the AG-derived EE with indirect calorimetry-measured EE.”*

**Methods**:

• Lines 80-84: With an N of 16 and 15/16 coming from one of two clinical trials where obesity was also an inclusion factor raises the question of why two clinical trials and why these data were pooled? That isn’t clear at present.

*Response: Both trials were conducted during overlapping periods at the same institution, with some participants involved in both trials. The primary purpose of pooling the two trials was to increase the overall sample size. To account for participants who participated in both trials, we treated their multiple visits as repeated measures across different time points since both trials used the same measurement protocols for cardiopulmonary exercise testing, resulting in 41 observations from 16 participants. After excluding data with invalid measures and accounting for repeated measures from overlapping participants, only 1 participant from one of the trials was finally included in this study. To maintain clarity and focus on research interest in the manuscript, we chose not to include detailed descriptions of this aspect, retaining only the essential information relevant to the study aims and outcomes.*

• Lines 96-97: this difference further brings up question as to why the data from the two trials were pooled, especially with the addition of only one additional participant.

*Response: We believe this question would be addressed in the previous response.*

• Lines 97-99: please consider moving this to the results.

*Response: We moved Table 1 to the results section.*

• Lines 102-104: please consider moving this after Lines 104-106 or integrate the two. This reads as if the protocol was ramped on METs but that is the response the actual ramp (speed and grade) was intended to achieve.

*Response: Valid point. We revised the sentences based on your comment. – lines 160-163: “The treadmill used a gradual ramping protocol beginning at a speed of 1.0 miles per hour (mph) and an incline of 0%, and every 30 seconds, the speed and incline were increased by 0.1 mph and 0.5%, respectively, leading to an approximate increase of 0.3 metabolic equivalents (METs) every 30 seconds [33, 34].”*

• Lines 109-111: If you eliminate the last 5% of the data, might that include the VO2peak data? This may be dependent on the interval these data are output in so please consider specifying that aspect. The two citations provided here are for steady state data which is considerably different from CPET data where a plateau isn’t likely so I’m not sure what the authors mean by variability below 5% for a plateau here.

*Response: We manually reviewed the initial raw data to determine an optimal range for data exclusion to minimize variability at the beginning and end of the test and retain a steady state during the CPET. We considered potential sources of variability that could not be controlled, such as participants holding onto the treadmill or accidentally stopping before clock time stopped which could disrupt accurate measure. We also confirmed that eliminating 5% of the data, which was approximately 30 seconds at both the start and end of the test, did not significantly affect individuals' VO₂peak measurements. To clarify, we revised the sentence. – lines 168-170: “After thoroughly reviewing each participant’s raw dataset, the first and last 5% of the data were eliminated before analysis and retained a steady state during CPET to minimize the variability from the potential uncontrollable sources [14, 36]”*

• Lines 122-125: The GT9X is not the latest generation of ActiGraph accelerometers and is in fact no longer in production by the manufacturer (<https://blog.theactigraph.com/blog/gt9x-link>) and there are several newer device generations currently available (<https://theactigraph.com/wearable-devices>).

*Response: Valid point. We revised the sentence to correct the fact that the GT9X is not the latest generation model of the AG accelerometer. – lines 181-184: “**The GT9X is one of the models of AG accelerometers, capable of measuring acceleration counts in three axes (X, Y (vertical on waist), and Z) within a dynamic range of ± 8 g, operating at sampling frequencies ranging from 30 to 100 Hz [12, 37]”*

• Line 124: the vertical, anteroposterior, and mediolateral orientations are generally used for devices worn on the waist, and while they also generally apply to the ankle, they should probably be listed as X, Y (vertical on waist), and Z.

*Response: Thank you for your valuable comment. We revised the sentence in accordance with your comment.*

• Lines 125-126: was the optional Idle Sleep Mode feature on or off for these data collections? It probably won’t have much bearing on these data since the authors use counts and not raw data but please consider specifying for transparency.

*Response*: The optional Idle sleep mode feature was turned off. We specified this in the *manuscript. – lines 184-185: “For the present study, the GT9X was initialized with a 60 Hz sampling frequency with the optional Idle Sleep Mode feature off.”*

• Lines 126-128: Please consider revising for clarity. The raw .gt3x files were downloaded and converted to counts in the specified epochs. Were these counts data converted from the raw using the normal filter or low frequency extension (LFE)? This is important for steps and counts so please specify.

*Response: This is a valid point. We revised the sentences to enhance clarity and added information about which filter was used to convert the data* format. We used the normal filter when converting raw acceleration data to *epoch data since moderate to high-intensity levels of PA are expected during CPET. – lines 185-189: “**Accelerometer data were downloaded in raw format as .gt3x files and then converted into 1-, 10-, and 60-second epochs data using the normal filter in ActiLife software v. 6.13.3 (ActiGraph Inc, Pensacola, FL, USA) to remove high-frequency noise while preserving the relevant signal. The data was saved as CSV files to match the CPET data with the start and end times of the exercise test*.”

• Lines 142-144: interesting. I’m not sure I’ve ever seen the Crouter two regression method split like this but I suppose it makes some sense in this case. I’d just make it clear that this isn’t generally the implementation of this method. I suggest the authors call it an adapted version of the method for clarity. The CV is used to determine which of the two equations are used and in this case you’re forcing this to be only 1 equation regardless of the counts magnitude or CV. To be fair, with only treadmill walking and running, that would be the likely outcome but this is brute forced into that equation and not determined as the developer intended.

*Response: We agree with your comments. We revised the manuscript using the adapted version of the refined crouter equation for walking/running activities. – lines 202-205: “As the study used a laboratory setting with CPET, the adapted version of the refined Crouter equation, specifically developed for walking/running (i.e., if the VT counts⋅10-second^-1^ >8, and the coefficient variation of the counts per 10 seconds is ≤10) was used in the current study [12, 14].”*

• Lines 149-151: I understand the need to do this in this study, but cut-point scaling is generally not recommended. This may warrant mention in the discussion/limitations so that is apparent to readers.

*Response: Thank you for your valuable suggestion. We added the limitations of using an adapted version of the refined crouter equation regarding cut-point scaling. – lines 522-528: “Among several different EE estimation equations, we used an adapted version of the refined Crouter equation, particularly developed for walking/running activities, using both 10-sec and 60-sec epoch data; yet this cut-point scaling may not adequately account for the magnitude or coefficient variation of the counts during the treadmill test. Further research is warranted to explore the use of coefficient variation in determining the most accurate refined Crouter equation for activities rather than relying on a single equation.”*

• Table 1: I’m not sure I understand the “b” superscript here. What does the Crouter two regression method have to do with steps counts? This method is for EE not step counting.

*Response: We revised Table 1’s footnote clearly. We intended to describe here that “the Refined Crouter equation used 10-sec epoch activity count data to estimate EE.”*

**Results**:

• Lines 207-208: out of curiosity, was the one male from the second clinical trial? If so, that further raises the point about pooling the data from the two trials for this analysis. The authors can’t generalize these findings as it is a nearly all female study and may be better presented that way.

*Response: The one male was part of the first clinical trial (participant #12 from the UFA trial), which involved 15 participants in our study. We acknowledged the limitation that the study findings cannot be generalized to the overall HF population due to the large proportion of female participants, and we discussed this limitation in the discussion/limitations section. – lines 506-510: “First, the majority of participants were female, with only one male included, which limits the generalizability of our findings to the entire HFpEF population. Future studies with large sample sizes and more equal distribution of both genders are required to enhance the generalizability of the results in this clinical population.”*

• Lines 208-209: please define NYHA in the text.

*Response: We included definitions of NYHA classification in the text. – lines 271-273: “The participants included both Whites and Blacks, with the majority classified as NYHA class II, which is a classification of HF determined by the New York Heart Association, class II indicating a patient has mild symptoms and slight limitations on PA”*

• Lines 230-234: I’m not sure how much value the correlation analysis adds. I think it would be generally expected these measures would correlate with the criterion measures.

*Response: As the primary aim of this study is to assess the agreement between AG-derived and criterion measures, we believe that confirming their strong correlation is also valuable. Moreover, we reanalyzed all study outcomes, taking into account the structure of repeated measures to address variations between and within subjects across multiple observations using mixed models. Therefore, we recalculated the correlation coefficient with bias-corrected 95% confidence intervals using the bootstrapping method. This approach could provide stronger evidence of correlations between measures at the individual level.*

• Table 3: I’m not sure I understand how the intensity-specific step counts were achieved. At what temporal resolution were these data aligned? There isn’t a clean temporal indication of the switch from LPA to MVPA as this may have happened mid-interval so this couldn’t be cleanly aligned at the 30s or 1-minute level, correct?

*Response: Valid point. To clarify, we further categorized PA intensity levels into LPA and MVPA using estimated EE obtained from indirect calorimetry, and these are described in the methods/statistical analysis section. Then, the total step counts associated with these intensity levels were summarized in Table 3. Your observation regarding the lack of a clear temporal indication for transitions between LPA and MVPA is valid, making it challenging to precisely align intensity levels with specific time points. Therefore, we decided to use the estimated EE derived from the criterion measure (indirect calorimetry) to define intensity levels according to the current PA guidelines and subsequently summarized the total step counts corresponding to those intensity levels.*

**Discussion**:
• Lines 335-336: please see also. This is why it is important to state whether your step counts come from the normal filter or LFE as that directly affects interpretation of these results.
o Toth, L. P., Park, S., Pittman, W. L., Sarisaltik, D., Hibbing, P. R., Morton, A. L., ... & Bassett, D. R. (2019). Effects of brief intermittent walking bouts on step count accuracy of wearable devices. Journal for the Measurement of Physical Behaviour, 2(1), 13-21.

*Response: We understand your valid point regarding the interpretation that can be affected by filters. LFE might be better for capturing low-frequency signals during activities with slow/less intense movements or irregular movement patterns. However, in our study, we employed a normal filter to remove noise while converting raw acceleration data into count data since a normal filter is well-suited for detecting the step counts in moderate-to-high intensity and regular movement patterns, which are generally expected during CPET. We specified what filter we used in the study in the methods section according to your earlier comments.*

• Lines 376-380: please change language about GT9X being the latest/newest/most recent ActiGraph device model.

*Response: We revised the terms of ‘latest/newest/most recent’ to ‘later/newer model’ to indicate GT9X to compare with the older AG model (model 7164).*

• Lines 447-450: please change language about GT9X being the latest/newest/most recent ActiGraph device model. Additionally, the GT3X devices were also triaxial devices. The prior GT1M and 7164 models were all uniaxial. Please revise.

*Response: Thanks for your clarification. We revised the term for GT9X as mentioned earlier, and also revised the model names to accurately reflect their uniaxial features. – lines 497-501: “Additionally, our study used the later generation of the AG, the GT9X model, which is equipped with triaxial sensors and improved algorithms capable of providing more comprehensive and accurate activity measurements than the earlier models (e.g., Model GT1M and 7164), which was limited to a uniaxial feature [12].”*

• Lines 470-471: The authors may have the opportunity in future work with these data to explore relative intensity with these patients since traditional absolute intensity measures may not be particularly helpful in the clinic or free-living evaluation of their physical behaviours. This may overcome some of the interpretation limitations the authors mention. Rowlands, A. V., Orme, M. W., Maylor, B., Kingsnorth, A., Herring, L., Khunti, K., ... & Yates, T. (2023). Can quantifying the relative intensity of a person’s free-living physical activity predict how they respond to a physical activity intervention? Findings from the PACES RCT. British Journal of Sports Medicine, 57(22), 1428-1434.
o Orme, M. W., Lloyd-Evans, P. H., Jayamaha, A. R., Katagira, W., Kirenga, B., Pina, I., ... & Rowlands, A. V. (2023). A case for unifying accelerometry-derived movement behaviors and tests of exercise capacity for the assessment of relative physical activity intensity. Journal of Physical Activity and Health, 20(4), 303-310.
o Kingsnorth, A. P., Rowlands, A. V., Maylor, B. D., Sherar, L. B., Steiner, M. C., Morgan, M. D., ... & Orme, M. W. (2022). A more intense examination of the intensity of physical activity in people living with chronic obstructive pulmonary disease: insights from threshold-free markers of activity intensity. International Journal of Environmental Research and Public Health, 19(19), 12355.

*Response: We appreciate your insightful suggestion and agree that exploring relative intensity could provide valuable insights into future research. We will consider these references in our future work to address the limitations noted in our current study.*

• Lines 473-474: the authors should consider mentioning as a limitation that the application in which these EE methods were applied is also not generally the intended use case having data from only a CPET instead of additional activities like those these methods were developed with.

*Response: Thank you for your valuable feedback. We added limitations based on your comment. – lines 530-539: “Given that a few EE estimation equations used in this study were originally developed for different types of activities (i.e., free-living activities), future research should incorporate a range of activities in addition to CPET and aim to explore the validity of the AG in real-life free-living conditions. Lastly, we could not consider the impact of absolute walking speed on the accuracy of the AG during exercise testing in this study due to the colinear increases in treadmill grade. Instead, we calculated the PA intensity level using EE estimation obtained during CPET and assessed the accuracy of AG at the corresponding PA intensity levels. Still, we must acknowledge that this approach is not specifically tailored for CPET data. Further investigations should examine the influence of absolute walking speed on the accuracy of the AG to provide a more comprehensive understanding of its validity across different exercise intensities during CPET.”*
