# Supplementary material for: Validity of the Actigraph-GT9X accelerometer for measuring steps and energy expenditures in heart failure patients
Source: PLoS One. 2024 Dec 30;19(12):e0315575. doi: 10.1371/journal.pone.0315575 (PMC11684600; doi:10.1371/journal.pone.0315575)
Supplement: Attachment — Submitted filename: R2_Response to Reviewers.docx [file pone.0315575.s007.docx]

Review Comments to the Author

**Reviewer #3**:

Methods:

• Lines 168-170: If you eliminate the last 5% of the data, might that include the VO2peak data? This may be dependent on the interval these data are output in so please consider specifying that aspect. The two citations provided here are for steady state data which is considerably different from CPET data where a plateau isn’t likely so I’m not sure what the authors mean by variability below 5% for a plateau here.

o Author Response: We manually reviewed the initial raw data to determine an optimal range for data exclusion to minimize variability at the beginning and end of the test and retain a steady state during the CPET. We considered potential sources of variability that could not be controlled, such as participants holding onto the treadmill or accidentally stopping before clock time stopped which could disrupt accurate measure. We also confirmed that eliminating 5% of the data, which was approximately 30 seconds at both the start and end of the test, did not significantly affect individuals' VO₂peak measurements. To clarify, we revised the sentence. – lines 168-170: “After thoroughly reviewing each participant’s raw dataset, the first and last 5% of the data were eliminated before analysis and retained a steady state during CPET to minimize the variability from the potential uncontrollable sources [14, 36]”

o **Reviewer Comment**: This remains unclear. With a ramped CPET to achieve VO2peak, steady state is not possible. This is why the test terminates at volitional fatigue because the participant cannot meet the demand.

- Thank you for your comment. While VO2 peak is indeed an important parameter, the primary focus of our study is on examining the validity of ActiGraph-GT9X for measuring step counts and estimating energy expenditure in comparison with the criterion. Regarding the CPET protocol, we did not stop the treadmill immediately when the participants reached volitional fatigue; instead, we gradually reduced the treadmill speed to allow participants to walk and recover. Therefore, to minimize potential bias and variability at the beginning and end of the data collection, we excluded 5% of the data from these phases. However, in response to the reviewer’s comment, we also provided additional results using raw data without excluding 5% of the data in the supplemental files. While there are slight variations in the numbers, we believe they do not substantially affect the study’s findings, interpretations, or overall implications.

**Reviewer #4**: The manuscript explores the validity of the ActiGraph-GT9X accelerometer for measuring step counts and energy expenditure in heart failure patients. The research topic is important given the clinical implications of accurate physical activity measurement in managing heart failure with preserved ejection fraction (HFpEF). While the study provides meaningful contributions, there are several areas where clarity, methodological rigor, and interpretation of results can be improved.

1. The sample’s gender imbalance (15 females, 1 male) limits generalizability. Discuss its impact and recommend future studies with more balanced gender representation.

- Thank you for your comment. We included the discussion on gender imbalance in the limitations section with the need for future research with large sample sizes and equal distribution of both genders.

- Lines 506-510: “First, the majority of participants were female, with only one male included, which limits the generalizability of our findings to the entire HFpEF population, particularly for male HF patients. Future studies with large sample sizes and more equal distribution of both genders are required to enhance the generalizability of the results in this clinical population.”

2. The Freedson and Sasaki equations overestimate EE at low PA levels. Further clarify how HFpEF characteristics may influence these discrepancies.

- The majority of the Actigraph-derived energy expenditure (EE) equations tended to under or overestimate across different PA intensity levels; however, the Sasaki equation showed better accuracy, particularly at low PA levels (mean bias=0.02; %bias=0.65 at LPA). Each equation estimated EE using algorithms based on acceleration data obtained from the triaxial accelerometer. Although some equations incorporate demographic information (i.e., Santos-Lozano VT/VM includes gender, and the Refined Crouter equation includes weight), particular HF characteristics are unlikely to be the primary factors influencing the acceleration data captured during the treadmill test across these equations. Instead, we provided potential explanations for the under or over-estimation of EE by each equation in the discussion sections along with evidence from previous literature (e.g., Freedson equation was developed using the older generation of AG (GT3X), Crouter equation was developed in the free-living settings, etc.) in the manuscript.

3. Speed variations were not accounted for, which may affect step count accuracy. Add discussion about the role of walking speeds, and recommend future studies involving real-world variability.

- Thank you for your comment. We included a discussion suggesting that future research should incorporate absolute walking speed to better account for real-world variability.

- Lines 533-540: Lastly, we could not consider the impact of absolute walking speed on the accuracy of the AG during exercise testing in this study due to the colinear increases in treadmill grade. Instead, we calculated the PA intensity level using EE estimation obtained during CPET and assessed the accuracy of AG at the corresponding PA intensity levels. Still, we must acknowledge that this approach is not specifically tailored for CPET data. Further investigations should explore the impact of absolute walking speed on the accuracy of the AG to better understand its validity across different exercise intensities during CPET, while considering real-world variability.

4. Provide more interpretation of MAPE and bias, particularly their clinical relevance for HFpEF patients. Discuss acceptable bias levels and their implications for step count and EE measurements.

- Thank you for your comment. Generally, MAPE of less than 10% is considered to indicate low bias, and we have included this threshold in our interpretation of ActiGraph-derived step counts and energy expenditure across different locations (for steps) and equations (for EE) with the references in the manuscript. Additionally, we provided interpretations for the percentage bias and Bland-Altman analysis, including mean bias and 95% limits of agreement, which help assess proportional bias within acceptable ranges and are described in Tables and Figures as well as in the manuscript. Although we did not establish specific cut-off values for acceptable bias ranges for each method in HFpEF patients, as we used various algorithms and approaches within a small sample size, we believe this study offers valuable clinical insights for researchers to choose the most appropriate device placement or energy expenditure equation for this population. We emphasized our study findings and the following clinical implications in the manuscript.
- Lines 369-371: The total SC obtained from the ankle-worn AG was significantly similar to manually counted steps and showed lower values of MAPE (<10%), percentage bias, and mean bias with narrow 95% LOA than the waist-worn AG, regardless of PA intensity level.
- Lines 550-555: Nonetheless, these findings offer valuable insight for future clinical research when selecting the most appropriate AG placement and EE prediction equations based on tailored to specific study populations and study settings. Furthermore, this study may provide crucial information guidance for utilizing ankle-worn AG to assess SC among HFpEF patients in clinical research settings where the established recommendations for AG placement are lacking. This will enhance the validity and reliability of PA assessment, contributing to the overall quality of future studies in this population.

5. Bland-Altman plots reveal proportional bias. Expand the discussion of the limits of agreement and clarify how proportional bias affects the accuracy of AG-derived measures.

- We included the interpretation for proportional bias obtained from Bland-Altman analysis in the manuscript. Overall, the majority of equations, except for the Freedson-Combined equation, had a significant proportional bias, leading to under- or over-estimation across the PA intensity levels. Although the Freedson-Combined equation showed no significant proportional bias, it still presented a wide 95% LOA range and a higher MAPE and percentage bias, indicating large variations and low accuracy compared to other equations.
- Lines 439-443: In the present study, the Freedson Combination equation was the only one that did not exhibit any proportional bias compared to indirect calorimetry, but it showed a wider 95% LOA range compared to other equations. Additionally, this equation still had the highest MAPE overall (28%) and at LPA (40%), with a more significant percentage bias than other equations, indicating large variations and low accuracy for EE estimation.

6. Using a standard resting EE (3.5 ml/kg/min) may not be appropriate for HFpEF patients. Emphasize this limitation and suggest using measured resting EE in future studies.

- Thank you for your comment. We included a discussion suggesting that further investigation should use measured resting EE.

- Lines 510-518: Another limitation is the reliance on standard resting EE (3.5 mL/kg/min) instead of measured resting EE for the calculation of METs values, which is considered a more accurate indicator of resting metabolic rate for the calculation of METs values [60]. A recent study found that the discrepancy between measured and estimated resting EE becomes more pronounced as BMI and age increase among individuals with HFpEF [61]. Considering our study population consists of adults with HFpEF whose mean BMI is greater than 38 and whose mean age is above 60 years, it is advisable to measure resting EE before the test to obtain more reliable results. Therefore, future studies should incorporate the measured resting EE to ensure the validity of the findings.
